# Efficient Removal of Co^2+^ from Aqueous Solution by 3-Aminopropyltriethoxysilane Functionalized Montmorillonite with Enhanced Adsorption Capacity

**DOI:** 10.1371/journal.pone.0159802

**Published:** 2016-07-22

**Authors:** Zhujian Huang, Pingxiao Wu, Beini Gong, Yaping Dai, Pen-Chi Chiang, Xiaolin Lai, Guangwei Yu

**Affiliations:** 1 College of Natural Resources and Environment, South China Agricultural University, Guangzhou 510642, China; 2 School of Environment and Energy, South China University of Technology, Guangzhou Higher Education Mega Centre, Guangzhou 510006, China; 3 The Key Lab of Pollution Control and Ecosystem Restoration in Industry Clusters, Ministry of Education, Guangzhou 510006, China; 4 Guangdong Provincial Engineering and Technology Research Center for Environmental Risk Prevention and Emergency Disposal, South China University of Technology, Guangzhou Higher Education Mega Centre, Guangzhou 510006, China; 5 Graduate Institute of Environmental Engineering, National Taiwan University, Taipei 106, Taiwan, China; Duke University Marine Laboratory, UNITED STATES

## Abstract

To achieve a satisfactory removal efficiency of heavy metal ions from wastewater, silane-functionalized montmorillonite with abundant ligand-binding sites (-NH_2_) was synthesized as an efficient adsorbent. Ca-montmorillonite (Ca-Mt) was functionalized with 3-aminopropyl triethoxysilane (APTES) to obtain the APTES-Mt products (APTES_1.0CEC_-Mt, APTES_2.0CEC_-Mt, APTES_3.0CEC_-Mt, APTES_4.0CEC_-Mt) with enhanced adsorption capacity for Co^2+^. The physico-chemical properties of the synthesized adsorbents were characterized by spectroscopic and microscopic methods, and the results demonstrated that APTES was successfully intercalated into the gallery of Ca-Mt or grafted onto the surface of Ca-Mt through Si-O bonds. The effect of solution pH, ionic strength, temperature, initial concentrations and contact time on adsorption of Co^2+^ by APTES-Mt was evaluated. The results indicated that adsorption of Co^2+^ onto Ca-Mt, APTES_1.0CEC_-Mt and APTES_2.0CEC_-Mt can be considered to be a pseudo-second-order process. In contrast, adsorption of Co^2+^ onto APTES_3.0CEC_-Mt and APTES_4.0CEC_-Mt fitted well with the pseudo-first-order kinetics. The adsorption isotherms were described by the Langmuir model, and the maximum adsorption capacities of APTES_1.0CEC_-Mt, APTES_2.0CEC_-Mt, APTES_3.0CEC_-Mt and APTES_4.0CEC_-Mt were 25.1, 33.8, 61.6, and 61.9 mg·g^-1^, respectively. In addition, reaction temperature had no impact on the adsorption capacity, while both the pH and ionic strength significantly affected the adsorption process. A synergistic effect of ion exchange and coordination interactions on adsorption was observed, thereby leading to a significant enhancement of Co^2+^ adsorption by the composites. Thus, APTES-Mt could be a cost-effective and environmental-friendly adsorbent, with potential for treating Co^2+^-rich wastewater.

## Introduction

Industries such as mining, electronics, metallurgy, electroplating and painting discharge large amounts of heavy metals and other hazardous substances daily into the soil and water environment. Heavy metal ions from industrial wastewater have attracted broad attention due to their toxicity and non-degradability, posing a huge threat to the ecological environment and human health. Cobalt is an essential trace element for the human body [1], but excessive amount of this element, which can cause paralysis, lung irritations, low blood pressure, and bone defects [2], is harmful to human health. Thus, treatment of cobalt-rich wastewater before it is discharged into the water environment is crucial.

Up till now, various methods have been developed and used to remove metal ions from wastewater, such as chemical precipitation, coagulation, electrochemical, and adsorption treatments [3–5]. Among these methods, adsorption is a widely applied and promising technology due to its high-efficiency and cost-effectiveness. Many literatures have reported different kinds of materials that could be used as adsorbents for heavy metal adsorption such as Co^2+^ adsorption, including bentonite, sepiolite, palygorskite, bagasse pith, cation exchange resin, and activated carbon. Among these materials and methods, cation exchange resin is quite effective [6, 7, 8], but the cost is expensive; activated carbon is inefficient for treating the wastewater with moderate and low concentrations of heavy metal [7, 9]. Low cost adsorbents including clay minerals, zeolites, chitosan, industrial waste products, and other agricultural wastes are efficient and have great potential for heavy metal adsorption [10–12]. Clay minerals with a large surface area and exchange capacity are an important constituent of soil and can efficiently adsorb metal ions. One of the clay minerals, montmorillonite, has been widely used for the treatment of heavy metal-contaminated wastewater [13], and many researchers have taken efforts to improve the adsorption of montmorillonite through various kinds of modification. Malakul *et al*. [14] and Krishna *et al*. [15] used surfactants to improve the adsorption of heavy metals onto montmorillonite. Inorganic modification were also extensively studied through the pillaring of montmorillonite by polyhydroxocations such as hydroxyl Al, hydroxyl Fe-, hydroxyl Zr and so on[16–18].

Recently, our group has developed a series of low-cost adsorbents or catalysts based on modified montmorillonite [16, 17, 19–25]. To develop an efficient adsorbent with plenty of ligand-binding sites (-NH_2_) for the treatment of Co^2+^-rich wastewater, a series of APTES-functionalized montmorillonites with different cation exchange capacities were prepared and their physicochemical properties were analyzed by XRD, FTIR, SEM and N_2_ adsorption-desorption. The adsorption kinetics and equilibrium of Co^2+^ onto APTES-functionalized montmorillonite were studied and the effects of pH, temperature and ionic strength on the adsorption were also investigated. Based on the above results, possible mechanisms of Co^2+^ adsorption onto APTES-functionalized montmorillonite were deciphered.

## Materials and Methods

### Materials and apparatus

Ca-montmorillonite (Ca-Mt) with a basal spacing of 1.59 nm and a cation exchange capacity (CEC) of 78 mmol 100 g^-1^ was used in this study. It consists of 32.4% of Si, 50.8% of O, 1.86% of Mg, 6.75% of Al, 0.09% of K, 1.7% of Ca, 2.07% of Fe, and 0.72% of Na [16].

All chemicals adopted in the study including HCl, NaOH, CoCl_2_·6H_2_O, and KNO_3_ are of analytical grade, and are purchased from Guangzhou Chemical Reagent Factory, Guangdong province, China. APTES was obtained from Aladdin Industrial, Shanghai.

Powder X-ray diffraction (XRD) of the materials was recorded using a powder diffractometer Bruker D8 ADVANCE at 40 kV and 20 mA with Cu Kα radiation. The Fourier-transform infrared (FTIR) spectroscopy of the products was measured by a FTIR spectrometer from 4000 to 400 cm^-1^ (American Thermo-electron Corporation). The measurement was carried out with a KBr pellet method (0.2% to 1% of the sample in KBr). The scanning electron microscopy (SEM) images of the obtained products were recorded by a S-3200N scanner, with accelerating current of 80 μA and voltage of 20 kV. Specific surface areas were determined by adsorption-desorption of nitrogen at 77 K using a Micromeritics ASAP 2020 surface area and porosity analyzer.

### Preparation of APTES-montmorillonites

Synthesis of APTES-montmorillonites was carried out by dispersing dried montmorillonite in cyclohexane at a ratio of 1:20 (*w/v*), with APTES further added. The suspension was mixed, then was heated and refluxed for 20 h at 60°C. APTES will hydrolyze with the surface of montmorillonite which is full of -OH groups. This process can also be called “grafting”, in which silanes are grafted on montmorillonite though hydrolyzation. The obtained products were separated by centrifugation at 4000 r·m^-1^ and then washed 7 times with anhydrous ethanol. The obtained samples were dried at 60°C overnight, ground to pass through a 200-mesh sieve. By adding different amounts of APTES during the synthetic procedure, APTES-montmorillonites with different cation exchange capacities were obtained, which were designated as APTES_1.0CEC_-Mt, APTES_2.0CEC_-Mt, APTES_3.0CEC_-Mt and APTES_4.0CEC_-Mt. The chemical stablilty of APTES-Mt is shown in **S1 Fig**.

### Batch adsorption experiments

Stock Co^2+^ solution was prepared by dissolving appropriate amount of CoCl_2_·6H_2_O in distilled water. Batch adsorption experiments were conducted under different conditions: neutral pH (6.8–7.5), room temperatures, initial Co^2+^ concentrations (10–300 mg·L^-1^), and contact time (0.5–36 h). The pH of Co^2+^ containing solution was adjusted by HCl (aq) and NaOH (aq). 0.05 g of the adsorbent was added into a 50 mL flask containing 25 mL of Co^2+^ containing solution, and the flask was agitated in water bath for a period of time. After that the mixture was centrifuged and atomic adsorption spectrometry (AAS) (Japan, Z-2000) was used to determine the concentration of Co^2+^ in the supernatant. To prevent any risk of metal contamination, all the flasks and tubes were presoaked in HNO_3_ for 24 h, washed strongly with distilled water and then dried in an oven. The desorption of Co^2+^ from Mt and APTES-Mt were determined. Mt and APTES-Mt after the adsorption experiment were mixed with 25.0 mL deionized water and agitated for 36 h to allow desorption of Co^2+^ to occur.

The adsorption capacity *q*_*e*_ (mg·g^-1^) is calculated according to the following equation.

$$q_{e}=\frac{V\;(C_{0}-C_{e})}{1000\;m}$$ (1)

Where *V* (mL) is the volume of Co^2+^ solution, *C*_*0*_ and *C*_*e*_ (mg·L^-1^) are the initial and equilibrium concentrations, respectively. *m* (g) is the mass of the adsorbent. All experiments were conducted in duplicate.

## Results and Discussion

### X-ray diffraction (XRD) and FTIR spectra of materials

**Fig 1:**
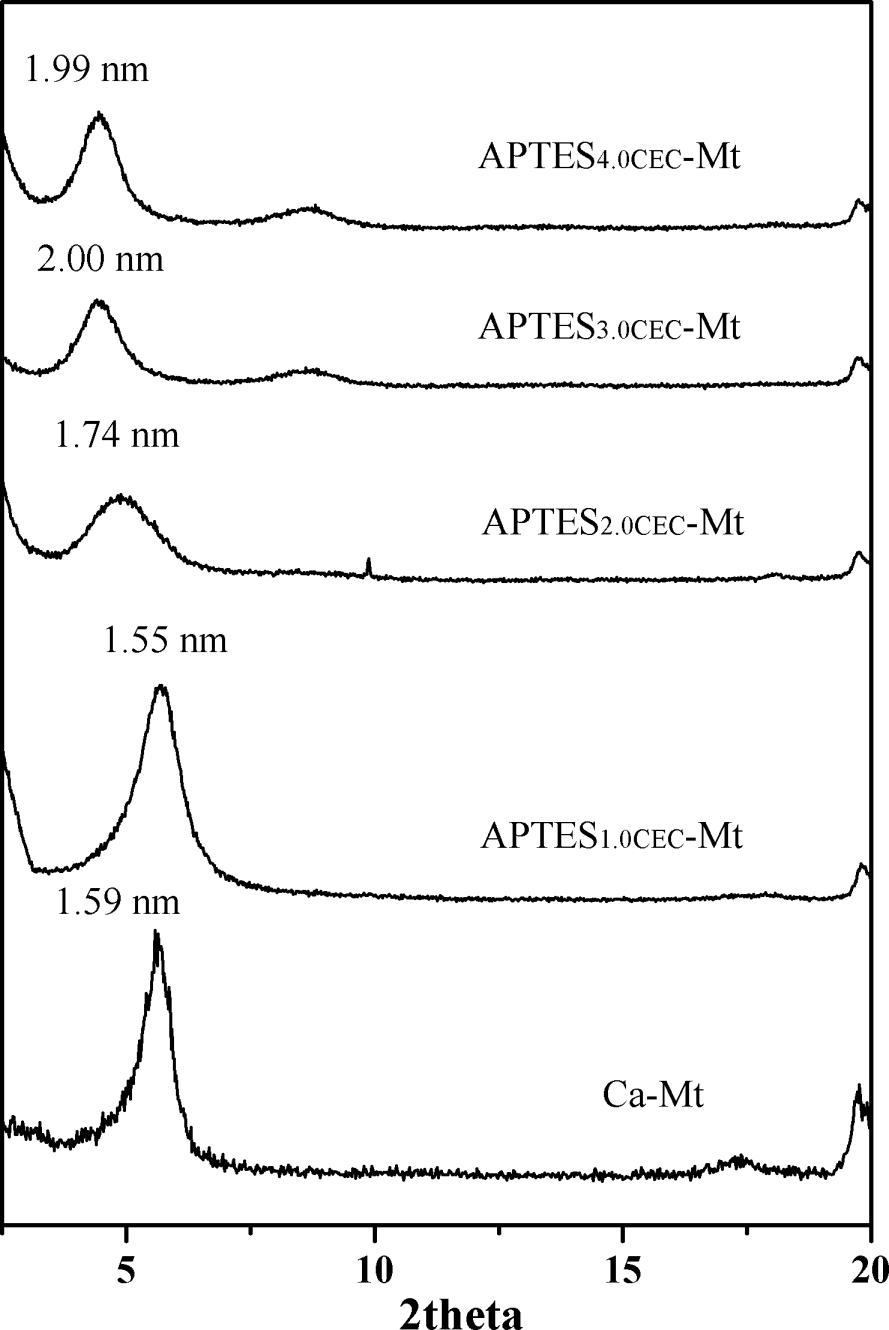
X-ray diffraction patterns of Ca-Mt, APTES_1.0CEC_-Mt, APTES_2.0CEC_-Mt, APTES_3.0CEC_- Mt and APTES_4.0CEC_-Mt.

XRD patterns of the pristine Ca-montmorillonite and APTES-Mts (**Fig 1**) showed that the (001) reflection intensity of Ca-montmorillonite was decreased after modification with APTES (APTES_2.0CEC_-Mt, APTES_3.0CEC_-Mt, APTES_4.0CEC_-Mt), demonstrating that disordered pillaring structure was formed. When a small amount of APTES molecules are intercalated between the layers of Mt, the basal spacing of the intercalated Mt were increased or even slightly reduced (APTES_1.0CEC_-Mt) due to interaction between the APTES molecules and the Mt backbone. But the d-spacing will eventually be increased when the intercalation amount is larger. It can be observed that the *d*-spacing is increased by 0.15 nm, 0.41 nm, and 0.40 nm for APTES_2.0CEC_-Mt, APTES_3.0CEC_-Mt, and APTES_4.0CEC_-Mt, respectively, which demonstrated that APTES was intercalated into the interlayer space of montmorillonite [20, 26].

**Fig 2:**
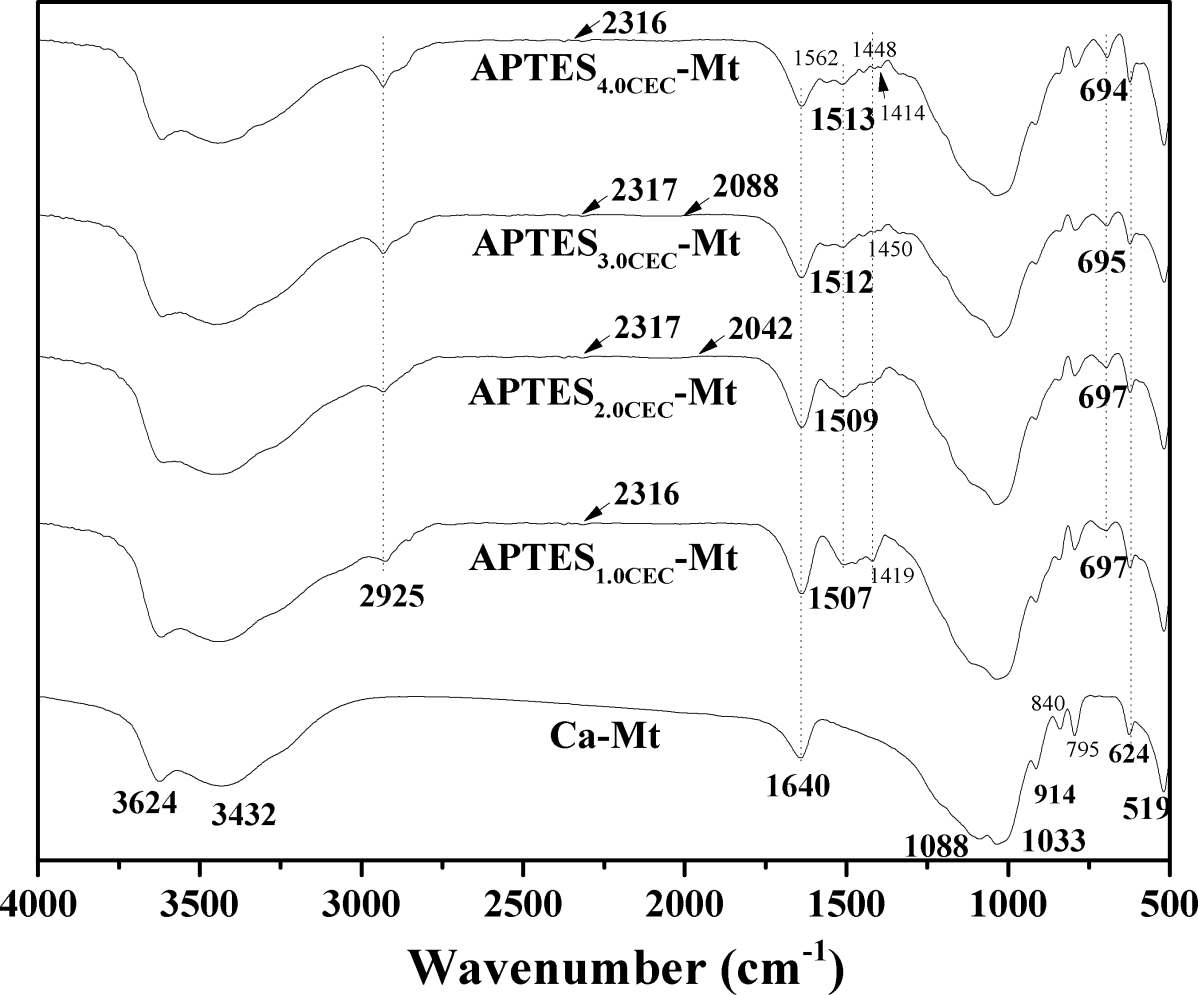
FTIR of Ca-Mt, APTES_1.0CEC_-Mt, APTES_2.0CEC_-Mt, APTES_3.0CEC_- Mt and APTES_4.0CEC_-Mt.

The FTIR spectra of Ca-Mt and APTES-Mts are presented in **Fig 2**, and the wavenumbers and assignment of peaks were listed in **S1 Table.** The bands of montmorillonite remained unchanged after modification, demonstrating that the basic crystal structure of Ca-Mt was not damaged. New bands appeared at 2925 cm^-1^, 2932 cm^-1^, and 2933 cm^-1^ could be ascribed to CH_2_ asymmetric stretching, indicating the existence of APTES in the obtained materials. The intensities of the bands increased from APTES_1.0CEC_-Mt to APTES_4.0CEC_-Mt, implying more APTES content on the modified montmorillonite with increased addition of APTES. Other new peaks exhibited at 1507 cm^-1^,1509 cm^-1^, 1512 cm^-1^, and 1513 cm^-1^ (N-H symmetric flexing), 1448 cm^-1^and 1450 cm^-1^ (CH_3_ asymmetric flexing), 1414 cm^-1^and 1419 cm^-1^ (C-H flexing), 694 cm^-1^, 695 cm^-1^, and 697 cm^-1^ (O-Si-O asymmetric flexing) [27], 2316 cm^-1^ and 2317 cm^-1^ (N-H stretching), 2042 cm^-1^ and 2088 cm^-1^ (NH_3_^+^ asymmetric stretching), 1562 cm^-1^ (-NH_3_^+^ symmetric flexing) indicated that APTES has been grafted on Ca-Mt. Similarly, the intensity of these bands also increased with increased addition of APTES.

### Surface and pore structure properties

**Fig 3:**
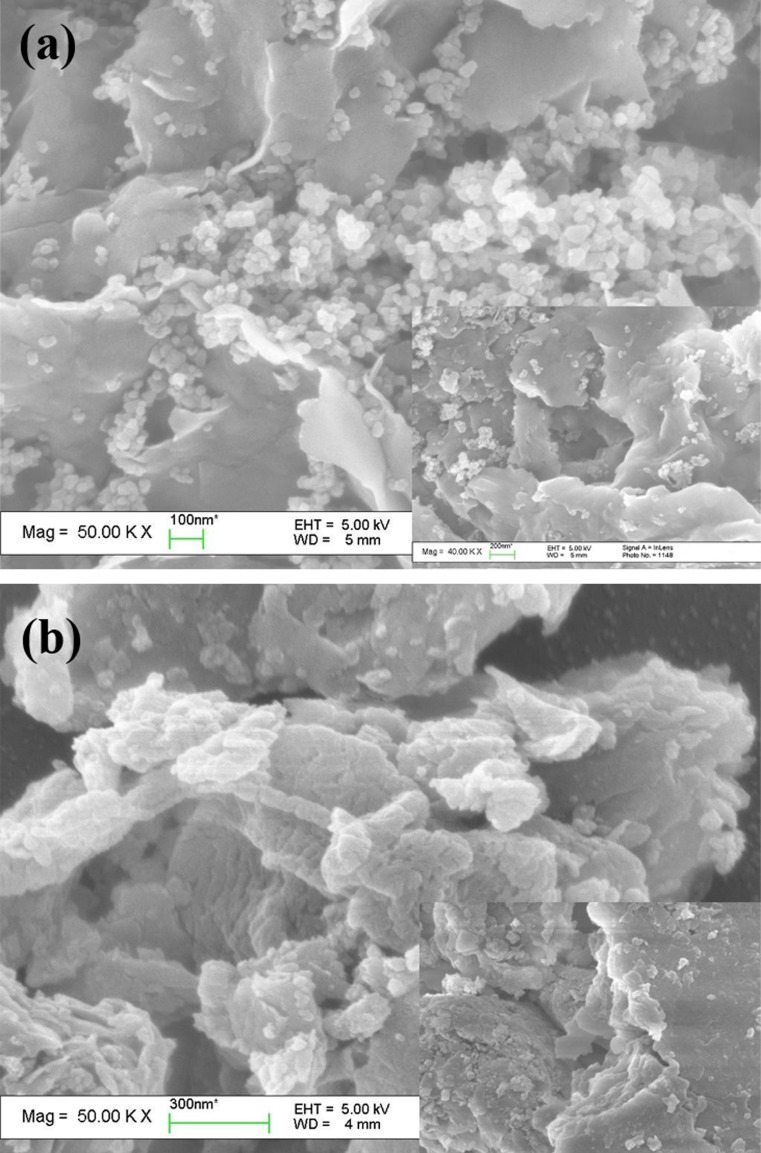
SEM images of Ca-Mt and APTES_3.0CEC_-Mt.

It can be observed in the SEM images that Ca-Mt was characterized with layered structure and smooth surface (**Fig 3A**). After APTES functionalization the layered structure was still apparent and unaltered, however, the surface was decorated with cracked lines and became rather uneven,which probably reflected surface functionalization with APTES (**Fig 3B**).

As was shown in **Table 1**, the BET surface area, exterior surface area and total volume of micropores of montmorillonite were reduced after functionalization with APTES, which probably resulted from APTES entering the interlayer space or micropores of montmorillonite. The pHzpc value of Ca-Mt was less than that of APTES_1.0CEC_-Mt, and the pHzpc values of APTES-Mt was increased with increased APTES content. The increase of pHzpc values of APTES-Mt compared with Mt further confirmed that positive charges have been introduced into Mt. And the gradual increase of the pHzpc values from APTES_1.0CEC_-Mt to APTES_4.0CEc_-Mt reflected increased APTES amounts in the composites.

**Table 1:** Point of zero charge, basal spacing and porosity of Ca-Mt and APTES-Mts.

Samples	*d*_001_ (nm)	pH_zpc_	S_BET_ (m^2^·g^-1^)	S_ext_ (m^2^·g^-1^)	D_a_ (nm)	V_t_ (cm^3^·g^-1^)
Ca-Mt	1.59	<1.0	71.15	51.90	13.629	0.1414
APTES_1.0CEC_-Mt	1.55	2.2	81.54	33.62	196.22	0.1056
APTES_2.0CEC_-Mt	1.74	6.0	16.48	14.17	290.56	0.0656
APTES_3.0CEC_-Mt	2.00	7.8	17.59	15.07	264.43	0.0643
APTES_4.0CEC_-Mt	1.99	8.3	11.91	9.94	341.42	0.0521

S_ext_ = external surface area, V_t_ = total porous volume, V_micro_ = microporous volume

**Fig 4:**
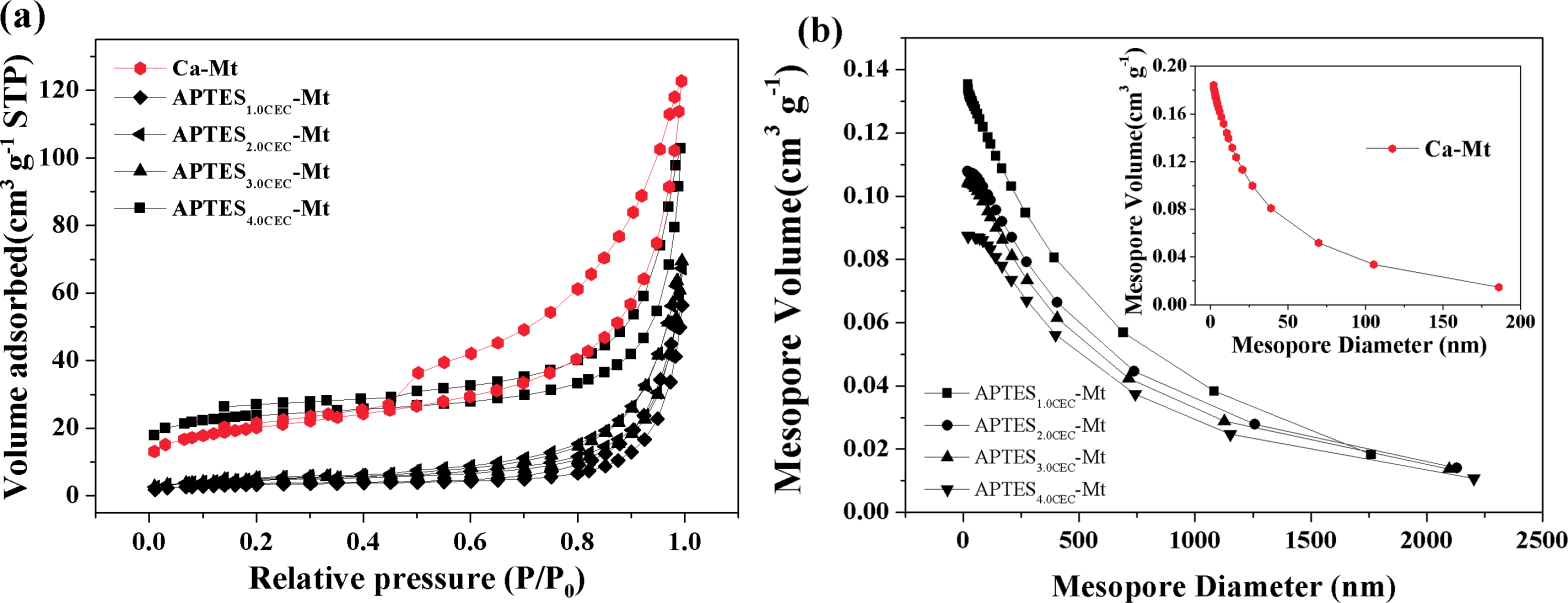
Nitrogen adsorption-desorption isotherms of Ca-Mt (a) and APTES-Mts (c); BJH pore size distribution of Ca-Mt (b) and APTES-Mts (d).

The nitrogen adsorption-desorption measurement was carried out and the results were presented in **Fig 4**. The hysteresis loops of all the APTES-Mts composites displayed steep adsorption and desorption branches at high P/P_0_ values and can be classified as type H3 loop. The composites were of IV adsorption-desorption isotherms, which indicated the mesoporous structure of the materials. The pore-size distribution of APTES-Mts composites showed that the mesoporous volumes were decreased after increasing the extent of functionalization. The nitrogen adsorption-desorption isotherms of APTES_2.0CEC_-Mt and APTES_3.0CEC_-Mt were similar compared with APTES_1.0CEC_-Mt, suggesting similar mesoporous structure except for APTES_1.0CEC_-Mt.

### Kinetic studies

**Fig 5:**
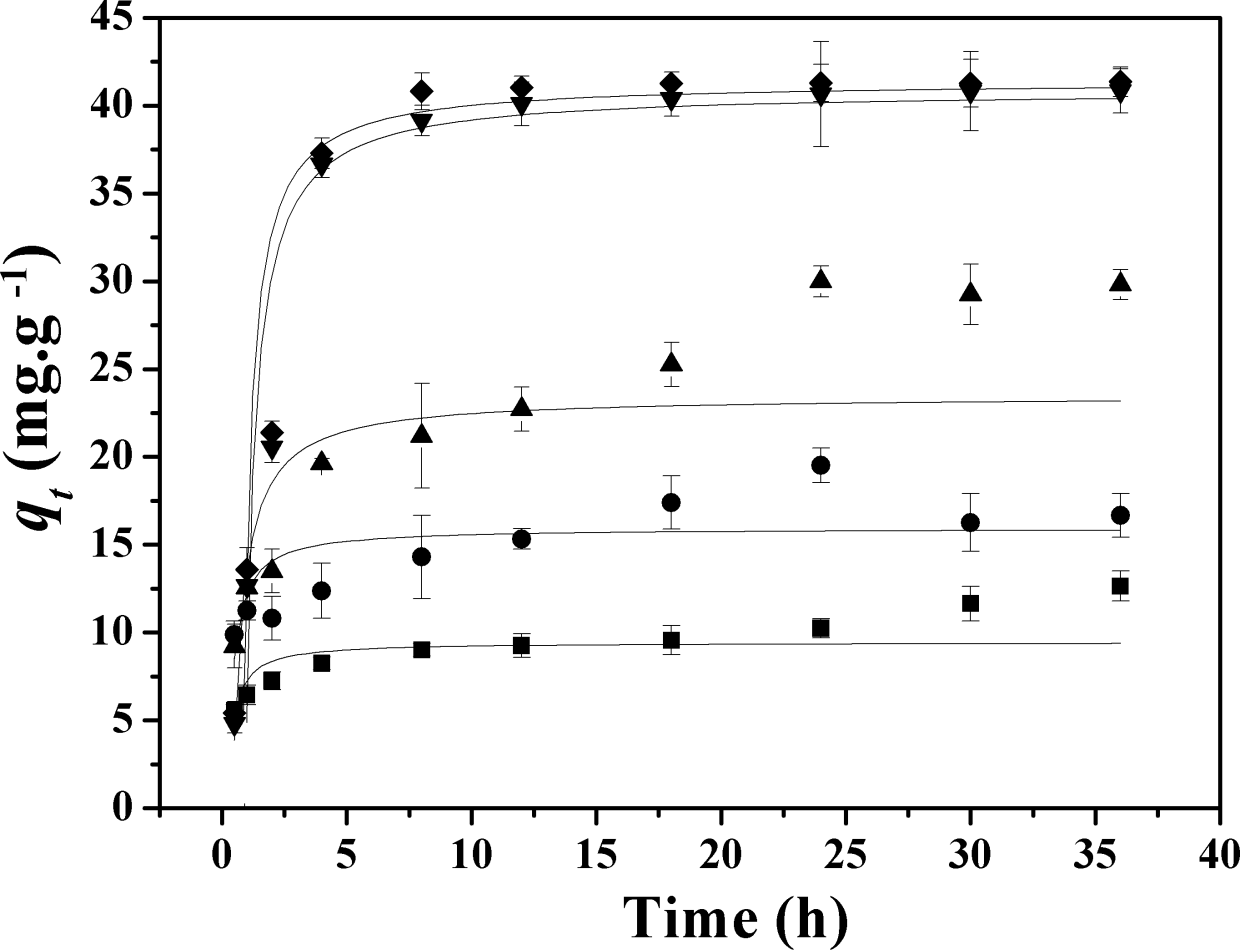
Effect of contact time on Co^2+^ adsorption by Ca-Mt and APTES-Mts: (■) Ca-Mt, (●) APTES_1.0CEC_-Mt, (▲) APTES_2.0CEC_-Mt, (▼) APTES_3.0CEC_- Mt, (◆) APTES_4.0CEC_-Mt.

**Fig 6:**
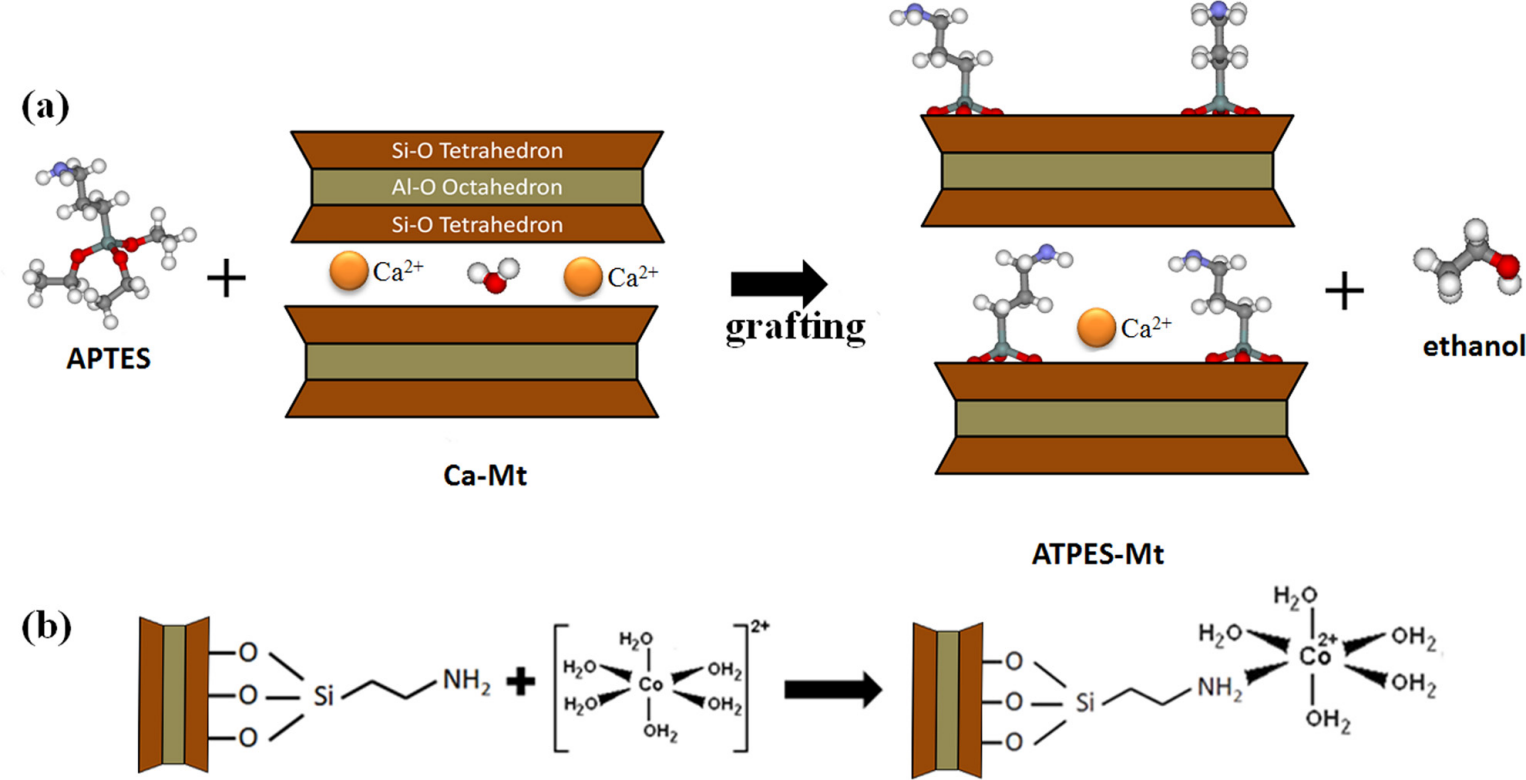
The synthetic procedure for APTES-Mt composite (a) and the cartoon illustration the coordination bond between Co(II) and APTES-Mt (b).

As was shown in **Fig 5**, the kinetic models revealed that the equilibrium was achieved after 8 h for APTES_3.0CEC_-Mt and APTES_4.0CEC_-Mt, while that for APTES_1.0CEC_-Mt and APTES_2.0CEC_-Mt was achieved after 30 h at pH 7, and the initial Co^2+^ consentration is 100 mg·L^-1^ (pH is controlled to make sure that Co^2+^ exists in ionic form in the aqueous solution). Generally, chemisorption or inner-sphere complexation of metal ions is fast while ion exchange or physical adsorption needs a longer time [6]. Accordingly, the adsorption of Co^2+^ onto APTES_1.0CEC_-Mt and APTES_2.0CEC_-Mt might be attributed to ion exchange or physical adsorption, and adsorption onto APTES_3.0CEC_-Mt and APTES_4.0CEC_-Mt was probably due to chemisorption or inner-sphere complexation. The synthetic procedure for APTES-Mt composite and the cartoon illustration of the coordination bond between APTES-Mt and Co(II) was displayed in **Fig 6** [7].

To better understand the adsorption kinetics, the adsorption data were analyzed using the pseudo-first-order (Eq (2)) and pseudo-second-order (Eq (3)) kinetic models. **Table 2** summarized the corresponding models fitting the parameters. As was shown in **Table 2**, the simulating data of Co(II) adsorption on Ca-Mt, APTES_1.0CEC_-Mt and APTES_2.0CEC_-Mt followed the pseudo-second-order kinetic expression, while adsorption by APTES_3.0CEC_-Mt and APTES_4.0CEC_-Mt fitted pseudo-first-order model well. The theoretical *q*_*e*_ values for Ca-Mt and APTES-Mts in the corresponding kinetic models are in good agreement with the experimental *q*_*e*_ values.

$$\text{ln}(\;q_{e}-q_{t})=\text{ln}\;q_{e}-k_{1}t$$ (2)

$$\frac{t}{q_{t}}=\frac{1}{k_{2}q_{e}^{2}}+\frac{t}{q_{e}}$$ (3)

Where *q*_*t*_ (mg·g^-1^) and *q*_*e*_ (mg·g^-1^) are the amounts of Co(II) adsorbed at time *t* (min) and at equilibrium, respectively; *k*_1_ (min^−1^) and *k*_2_ (g·mg^-1^ min^−1^) are the adsorption rate constants of the pseudo-first-order model and pseudo-second-order model, respectively.

**Table 2:** The kinetic parameters of adsorption by Ca-Mt and APTES-Mts.

		Pseudo-first-order model			Pseudo-second-order model		
Samples	*q*_*e*(experiment)_ (mg·g^-1^)	*K*_1_ (min^−1^)	*q*_*e*_ (mg·g^-1^)	*R*^2^	*K*_2_ (g·mg^-1^ min^−1^)	*q*_*e*_ (mg·g^-1^)	*R*^2^
Ca-Mt	12.02	0.88	9.82	0.65	0.18	10.24	0.89
APTES_1.0CEC_-Mt	16.67	1.27	15.71	0.38	0.11	16.72	0.65
APTES_2.0CEC_-Mt	29.82	0.41	26.65	0.78	0.02	29.53	0.90
APTES_3.0CEC_-Mt	40.84	0.40	40.94	0.98	0.01	45.34	0.94
APTES_4.0CEC_-Mt	41.37	0.41	41.73	0.98	0.01	46.07	0.94

**Fig 7:**
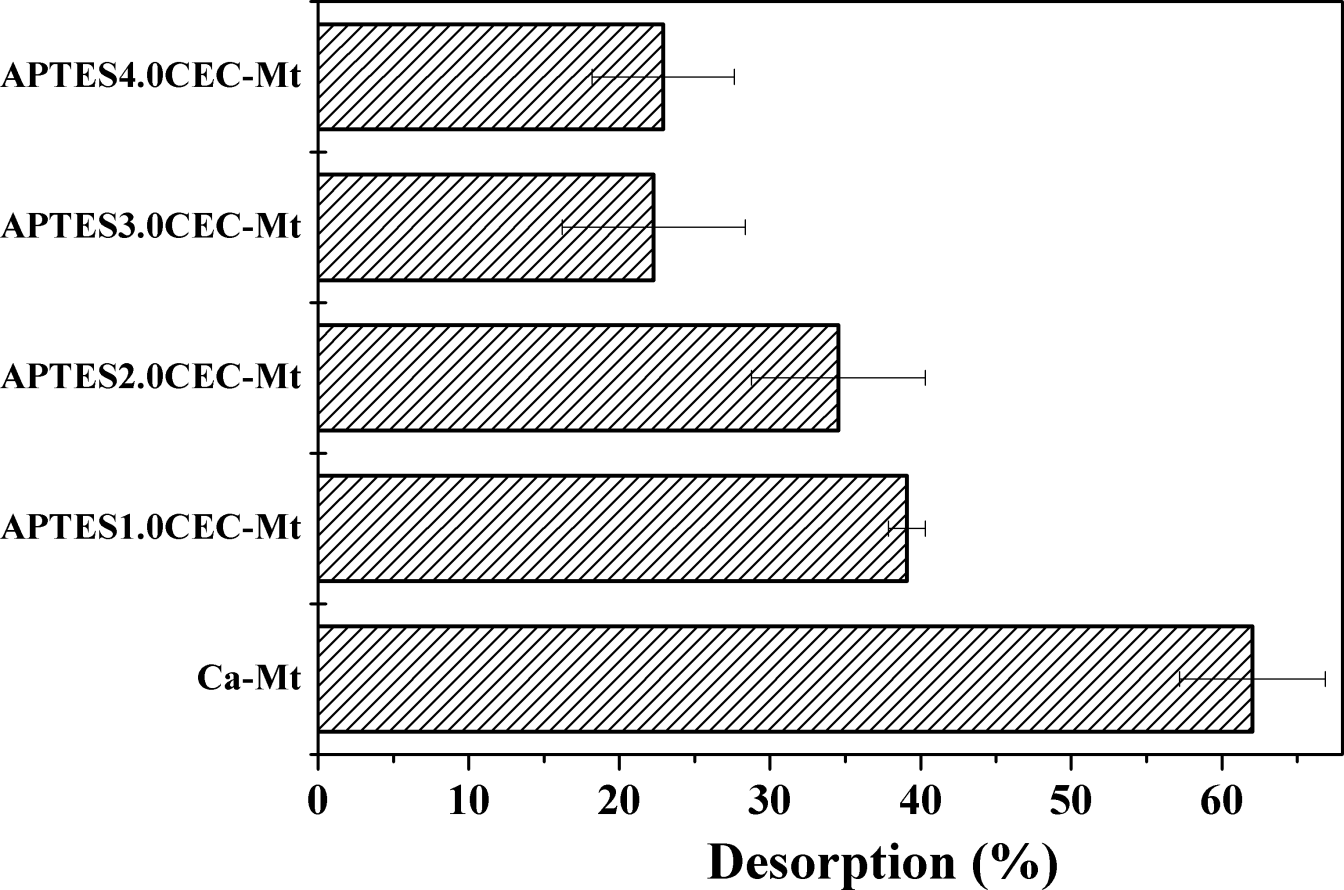
Desorption of Co^2+^ as related to their adsorption by Ca-Mt or APTES-Mts.

The desorption results were presented in **Fig 7**. It was found that more than 60% of Co^2+^ was desorbed from Ca-Mt within 36 h. However, Co^2+^ adsorbed on APTES-Mt was less likely to be desorbed. As the content of APTES increased, less Co^2+^ was desorbed from APTES-Mt. The adsorption of Co^2+^ on Ca-Mt is mainly attributed to ion exchange and coordination interaction. It will be more difficult to desorb heavy metals complexed with adsorbents in deionized water.

### Isotherm studies

**Fig 8:**
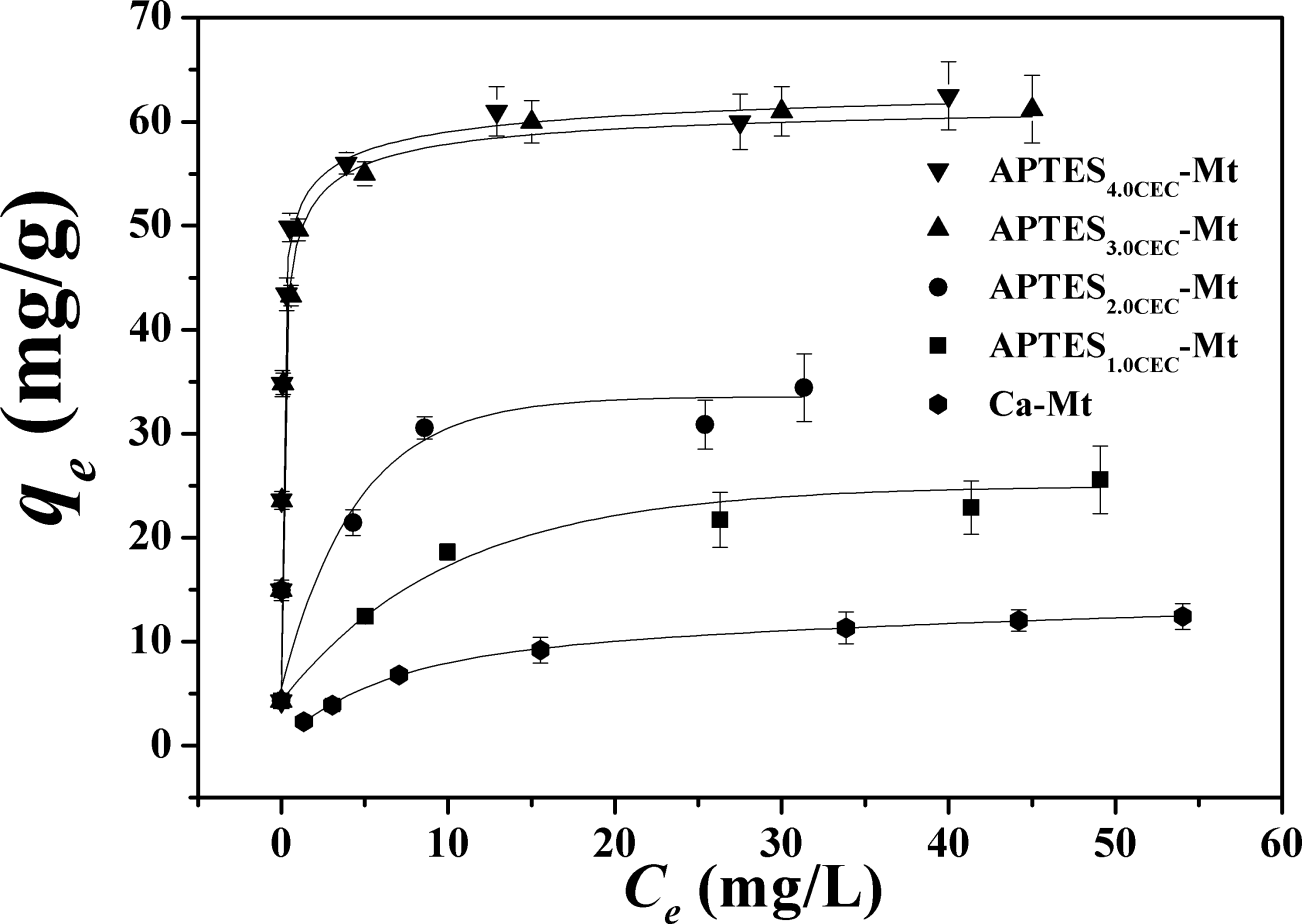
The adsorption isotherm of Co^2+^ by APTES-Mts.

Freundlich (Eq (4)) and Langmuir (Eq (5)) equations were used to analyze the adsorption data, and the fitting results were listed in **Table 3**. As evidenced by the correction coefficient *R*^2^, the Langmuir model could describe the adsorption process better than Freundlich model with *R*^2^ > 0.90, which was also the case with adsorption of heavy metals by organic montmorillonite in our previous study [23]. The adsorption isotherm of Co(II) by Ca-Mt and APTES-Mts and their corresponding Langmuir fitting curves were depicted in **Fig 8**. This isotherm fitting result indicated that the adsorption of Co(II) is likely a monolayer adsorption process.

$$q_{e}=K_{F}C_{e}^{n}$$ (4)

$$q_{e}=\frac{q_{e}K_{L}C_{e}}{1+K_{L}C_{e}}$$ (5)

Where *q*_*e*_ (mg·g^-1^) is the adsorption capacity at equilibrium, *q*_*m*_ (mg·g^-1^) stands for the maximum adsorption capacity, *K*_*F*_ (mg^1-1/n^ L^1/n^ g^-1^) and *n* are Freundlich isotherm constants; *K*_*L*_ (L·mg^-1^) is the Langmuir isotherm constant.

**Table 3:** Equilibrium isotherm model parameters for Co^2+^ adsorption onto APTES-Mts.

	Freundlich			Langmuir		
Samples	*K*_*F*_ (mg^1-1/n^ L^1/n^ g^-1^)	*n*	*R*^*2*^	*K*_*L*_ (L·mg^-1^)	*q*_*m*_ (mg·g^-1^)	*R*^*2*^
Ca-Mt	2.36	0.44	0.98	0.15	13.07	0.99
APTES_1.0CEC_-Mt	9.22	0.27	0.98	0.29	25.91	0.98
APTES_2.0CEC_-Mt	19.68	0.16	0.81	1.16	33.76	0.99
APTES_3.0CEC_-Mt	36.85	0.22	0.82	5.22	61.35	0.99
APTES_4.0CEC_-Mt	41.16	0.19	0.73	7.25	61.88	0.99

The adsorption capacity *q*_*e*_ (mg·g^-1^) increased in the following order: APTES_1.0CEC_-Mt < APTES_2.0CEC_-Mt < APTES_3.0CEC_-Mt ≈ APTES_4.0CEC_-Mt. The increased adsorption capacity could be explained as: For APTES_1.0CEC_-Mt, the basal spacing (*d*_001_) is equal to that of pristine montmorillonite and ion exchange is one important adsorption mechanism involved. There is a slight increase in basal spacing of APTES_2.0CEC_-Mt, although the physical adsorption of Co^2+^ onto the surface of montmorillonite is still the dominant mechanism. As for APTES_3.0CEC_-Mt and APTES_4.0CEC_-Mt, it’s apparent that APTES has entered into the layers, exchanged with Ca^2+^ and weakened physical adsorption, whereas chemisorption complexation (mainly coordinating adsorption) led to an noticeable increase in adsorption capacity, which is consistent with that reported for Sr^2+^ adsorption on APTES-Mts [20].

### Effect of solution pH and temperature

**Fig 9:**
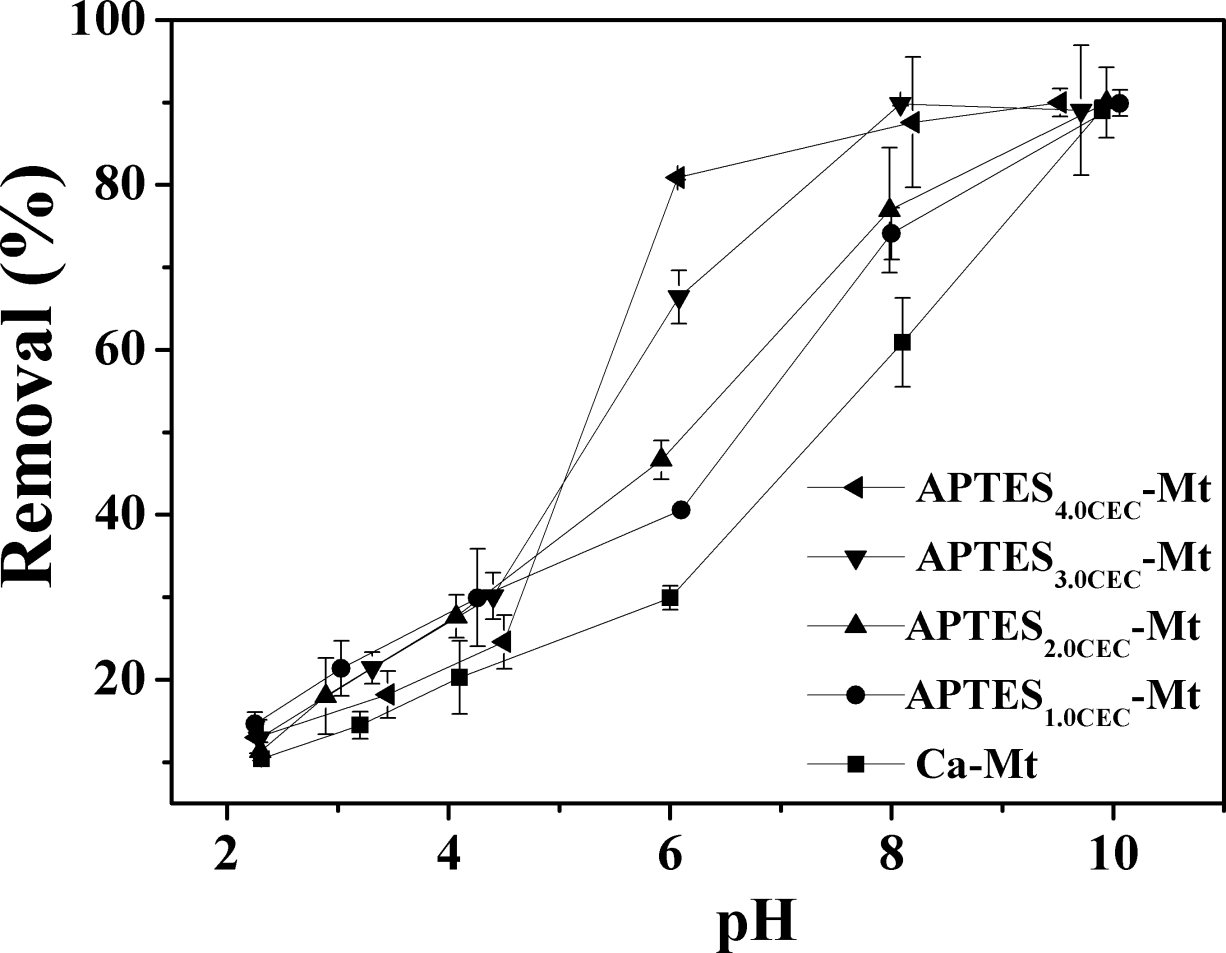
Effect of solution pH on the adsorption of Co^2+^ by APTES-Mts.

**Fig 10:**
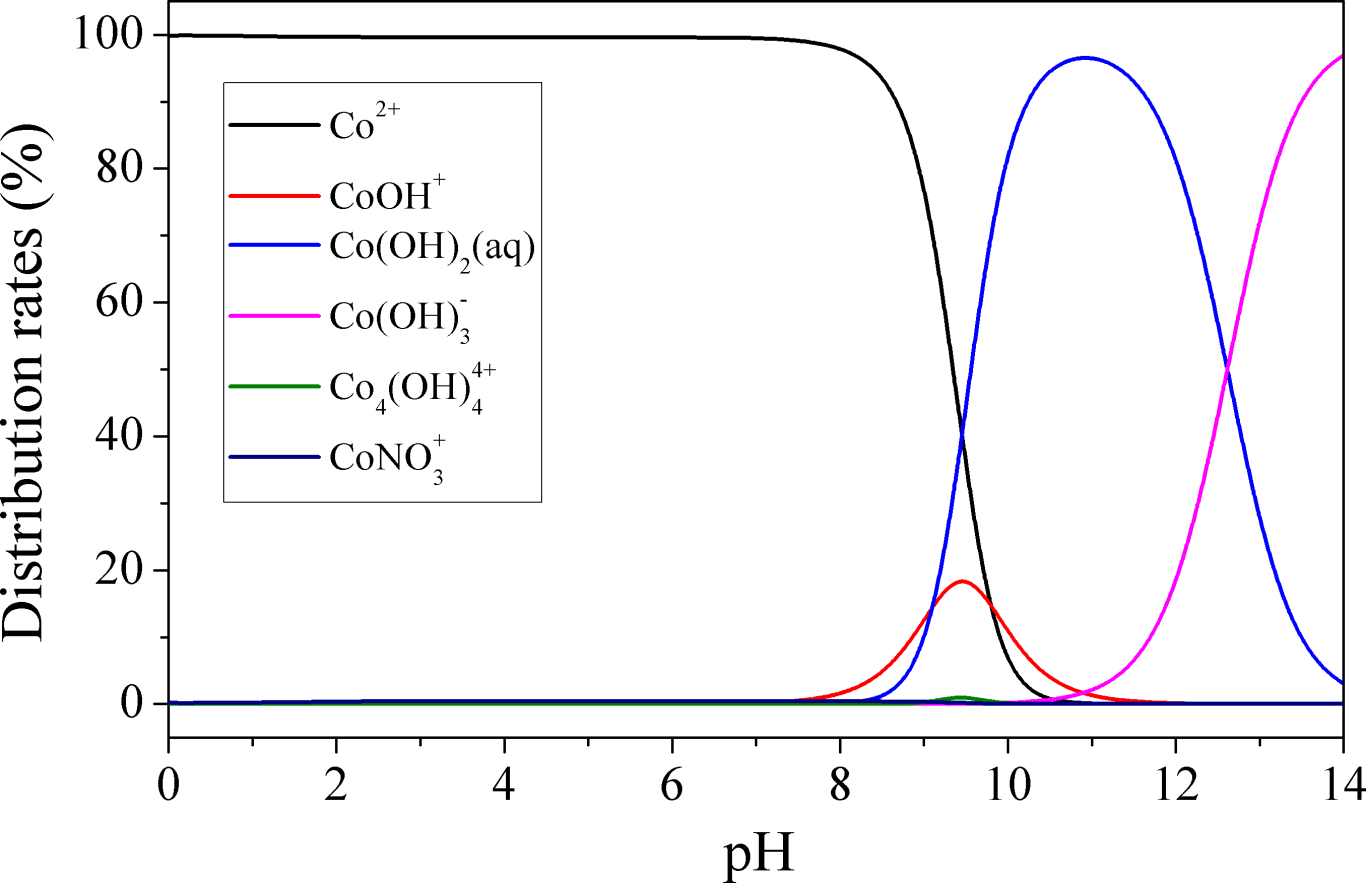
Distribution of Co(II) species under different pH values.

The removal of Co^2+^ under various pH conditions was determined. As was shown in **Fig 9**, the removal of Co^2+^ at pH values ranging from 2.0 to 10.0 revealed that the adsorption is significantly pH-dependent and the uptake of Co^2+^ increased with increased pH. Generally, the existing form of Co(II) and the surface charge of an adsorbent would be influenced by the solution pH. The possible form of Co(II) at different pH values (100 mg·L^-1^, 28°C) was calculated using the program visual MINTEQ, and the results was shown in **Fig 10**. The results indicated that the predominant Co(II) species is Co^2+^ (>95%) at pH < 7.5, while at pH > 7.5 the Co(II) species are present as Co^2+^, Co(OH)^+^, Co(OH)_2_(aq), Co(OH)_3_^−^, Co_4_(OH)_4_^4+^, and CoNO_3_^+^. At pH > 8.5, precipitation of cobalt hydroxide would occur. Additionally, the content of CoOH^+^ and Co(OH)_2_(aq) would increase above pH 8.5 [28]. Therefore, the discrepancies of adsorption capacities at pH 2~8 were attributed to adsorption, and the concentration of Co^2+^ would greatly decrease at pH > 8.5, which is mainly caused by cobalt hydroxide precipitation. At pH values ranging from 2.0 to 8.5, the influence of H^+^ on adsorption could be summarized as follows: At lower pH, excessive H^+^ ions would successively occupy the binding sites and compete with Co^2+^, leading to a low adsorption capacity for Co^2+^ [29]. Moreover, ligand-binding -NH_2_ groups of APTES on Ca-Mt would bond with H^+^ to form -NH_3_^+^ at acidic pH conditions, thus the coordination of -NH_2_ with Co^2+^ cations would be weakened. Under the experimental conditions, the adsorption capacity of APTES_3.0CEC_-Mt and APTES_4.0CEC_-Mt were larger than that of APTES_1.0CEC_-Mt and APTES_2.0CEC_-Mt. The adsorption reactions in the solutions are shown as follows:

$$\text{Ca-Mt}+{2\text{H}}^{+}\to \text{H-Mt-H}+{\text{Ca}}^{2+}$$ (6)

$$\text{Ca-Mt}\;+\;{\text{Co}}^{2+}\to \text{Co-Mt}\;+\;{\text{Ca}}^{2+}$$ (7)

$$\text{Mt}\equiv {\text{O}}_{3}\text{Si-O}-{\left({\text{CH}}_{2}\right)}_{2}{-\text{NH}}_{2}+\;{\text{H}}^{+}\to \text{Mt}\equiv {\text{O}}_{3}\text{Si-O}-{\left({\text{CH}}_{2}\right)}_{2}{-\text{NH}}_{3}^{+}$$ (8)

$$\text{Mt}\equiv {\text{O}}_{3}\text{Si-O}-{\left({\text{CH}}_{2}\right)}_{2}{-\text{NH}}_{2}+\;{\text{Co}}^{2+}\to \text{Mt}\equiv {\text{O}}_{3}\text{Si-O}-{\left({\text{CH}}_{2}\right)}_{2}{-\text{NH}}_{2}{-\text{Co}}^{2+}$$ (9)

**Fig 11:**
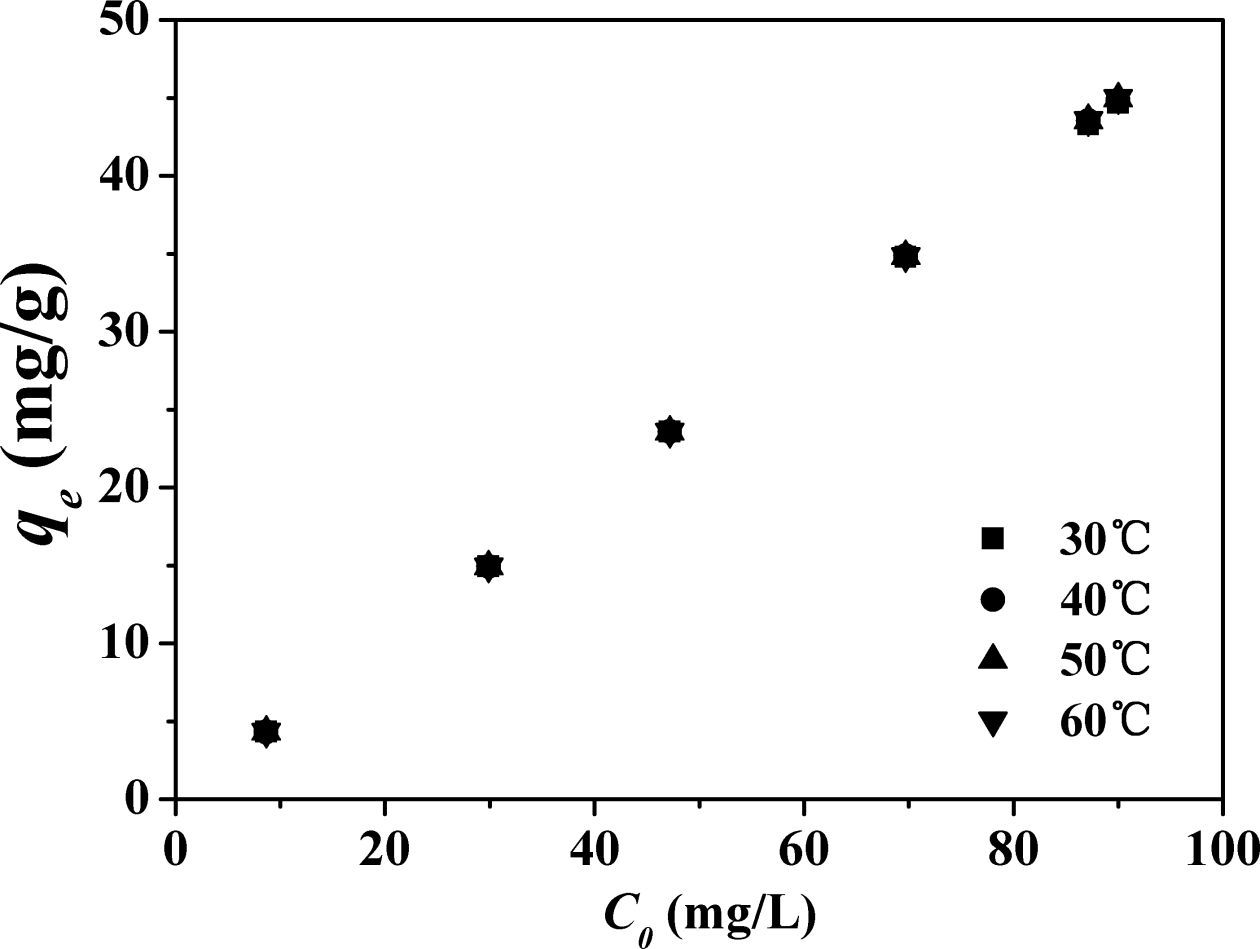
Effect of temperature on the adsorption of Co^2+^ by APTES_3.0CEC_-Mt.

The effect of temperature on adsorption of Co^2+^ onto APTES-Mts was also investigated. Adsorption of Co^2+^ onto APTES_3.0CEC_-Mt (as a representative) at 30°C, 40°C, 50°C, and 60°C was determined. As presented in **Fig 11**, it was found that the effect of temperature on the adsorption is negligible.

### Effect of electrolyte ionic strength

**Fig 12:**
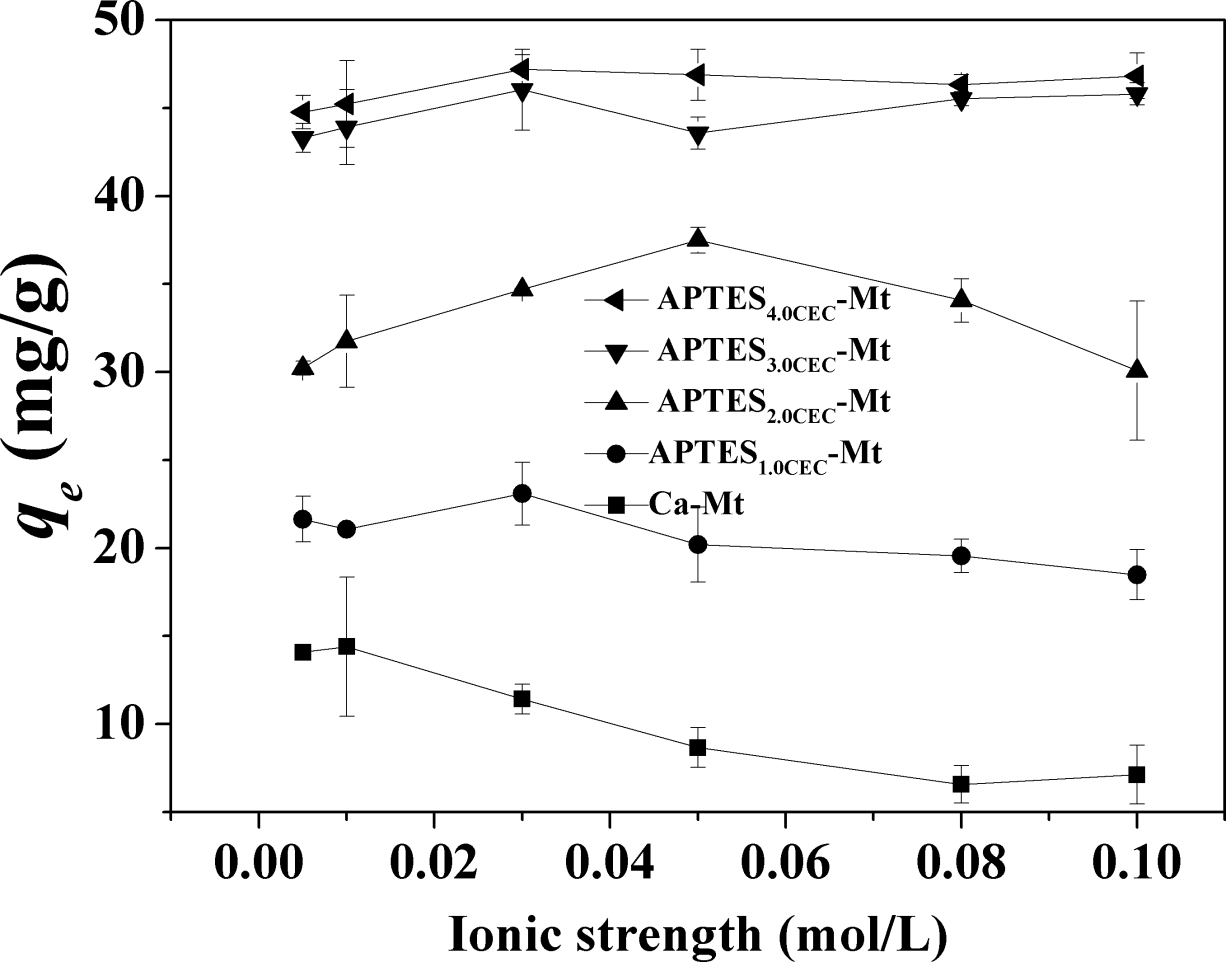
Effect of ionic strength (K^+^) on the adsorption of Co^2+^ by APTES-Mts.

Electrolyte ionic strength is one of the most critical factors that might influence the adsorption process [17, 30]. The ionic strength of the adsorption reaction was set at 0.005 mol·L^-1^, 0.01 mol·L^-1^, 0.03 mol·L^-1^, 0.05 mol·L^-1^, 0.08 mol·L^-1^, and 0.1 mol·L^-1^ with KNO_3_. As was presented in **Fig 12**, ionic strength of the solution exhibited little influence on the adsorption of Co^2+^ by APTES_3.0CEC_-Mt and APTES_4.0CEC_-Mt. As the ionic strength increased, the adsorption capacity increased at first and then showed a decrease for APTES_2.0CEC_-Mt, while adsorption by APTES_1.0CEC_-Mt actually decreased. These changes are related to the exchangeable ions in the galleries of the materials. For APTES_1.0CEC_-Mt, ion exchange is important for adsorption, and Ca^2+^ are the major cations that exchanged with Co^2+^. K^+^ in the solution would compete with Co^2+^ to exchange with Ca^2+^ and cause a decrease of Co^2+^ uptake. For APTES_2.0CEC_-Mt, the influence of ionic strength is weakened with reduced reactive sites of Ca^2+^ as a certain amount of APTES has entered into the layers of montmorillonite and replaced the Ca^2+^ cations, which was evidenced by the XRD and FTIR results, As for APTES_3.0CEC_-Mt and APTES_4.0CEC_-Mt, adsorption of Co^2+^ was mainly attributed to chemisorption or coordination, hence the solution ionic strength exerted little influence on Co^2+^ adsorption. The adsorption capacity and equilibrium time of APTES-Mt are compared with those of other adsorbents for removal of Co^2+^ reported in previous literatures (**S2 Table**). It can be observed that APTES-Mt has a relatively high capacities for the removal of Co^2+^ and an acceptably short reaction time, suggesting promising potential for the treatment of Co^2+^-rich wastewater.

## Conclusions

APTES functionalized montmorillonites with different cation exchange capacities were synthesized and employed for the adsorption of Co^2+^ from aqueous solution. Characterization of the obtained products demonstrated that APTES could be successfully intercalated into the interlayer space of Ca-Mt and grafted onto Ca-Mt, and connected with Si-O bindings within the silica tetrahedron plates. A series of batch adsorption experiments showed that the adsorption of Co^2+^ onto APTES-Mts was significantly influenced by the pH of the solution in the range of 2.0 to 8.0; however, the effect of pH was not significant if the pH value was higher than 8.0. Adsorption kinetics of Co^2+^ onto APTES_3.0CEC_-Mt and APTES_4.0CEC_-Mt could be well described by pseudo-first-order model, while adsorption onto APTES_1.0CEC_-Mt and APTES_2.0CEC_-Mt fitted the pseudo-second-order model. Langmuir adsorption isotherms could provide a well correlation for the adsorption of Co^2+^ onto APTES-Mts. The reaction temperature exhibits negligible influence on the adsorption process, and the adsorption of Co^2+^ on APTES_1.0CEC_-Mt and APTES_2.0CEC_-Mt was independent of the ionic strength of the solution. APTES could affect the surface properties of Ca-Mt, and provide ligand-binding sites to enhance the adsorption of heavy metals. Furthermore, ion exchange is the primary mechanism for Co^2+^ adsorption onto the APTES_1.0CEC_-Mt and APTES_2.0CEC_-Mt, while coordinate interaction was mainly accountable for the adsorption of Co^2+^ onto APTES_3.0CEC_-Mt and APTES_4.0CEC_-Mt. These preliminary results indicate that APTES functionalized montmorillonite should be a cost-effective, chemically-stable and environmental-friendly adsorbent for the treatment of Co(II)-rich wastewater.

## Supporting Information

S1 Fig **(a) The absorbance as a function of molarconcentrations of APTES, (b) The dissolved total nitrogen concentration under different pH values.** The chemical stability of APTES-Mt in different pH values was test by analyzing of the dissolved N. The stability of APTES-Mt is satisfied.
(DOC)

S1 Table Infrared wavenumbers and assignments of Ca-Mt and APTES-Mts. (DOC)

S2 Table Comparison of adsorption capacity of Co^2+^ on various adsorbents. (DOC)

S3 Table The raw data of adsorption of Co^2+^ in 30°C for better understanding of Fig 11. (DOC)
