# Correction: Efficient Removal of Co^2+^ from Aqueous Solution by 3-Aminopropyltriethoxysilane Functionalized Montmorillonite with Enhanced Adsorption Capacity

**DOI:** 10.1371/journal.pone.0164219

**Published:** 2016-09-29

**Authors:** Zhujian Huang, Pingxiao Wu, Beini Gong, Yaping Dai, Pen-Chi Chiang, Xiaolin Lai, Guangwei Yu

In the Funding section, the grant numbers from the National Science Foundation of China are listed incorrectly. The correct grant numbers are: 41273122, 41472038, 51509093.
