# Supplementary material for: Efficient Removal of Co2+ from Aqueous Solution by 3-Aminopropyltriethoxysilane Functionalized Montmorillonite with Enhanced Adsorption Capacity
Source: PLoS One. 2016 Jul 22;11(7):e0159802. doi: 10.1371/journal.pone.0159802 (PMC4957767; doi:10.1371/journal.pone.0159802)
Supplement: S1 Fig — (a) The absorbance as a function of molarconcentrations of APTES, (b) The dissolved total nitrogen concentration under different pH values. The chemical stability of APTES-Mt in different pH values was test by analyzing of the dissolved N. The stability of APTES-Mt is satisfied. (DOC) [file pone.0159802.s001.doc]

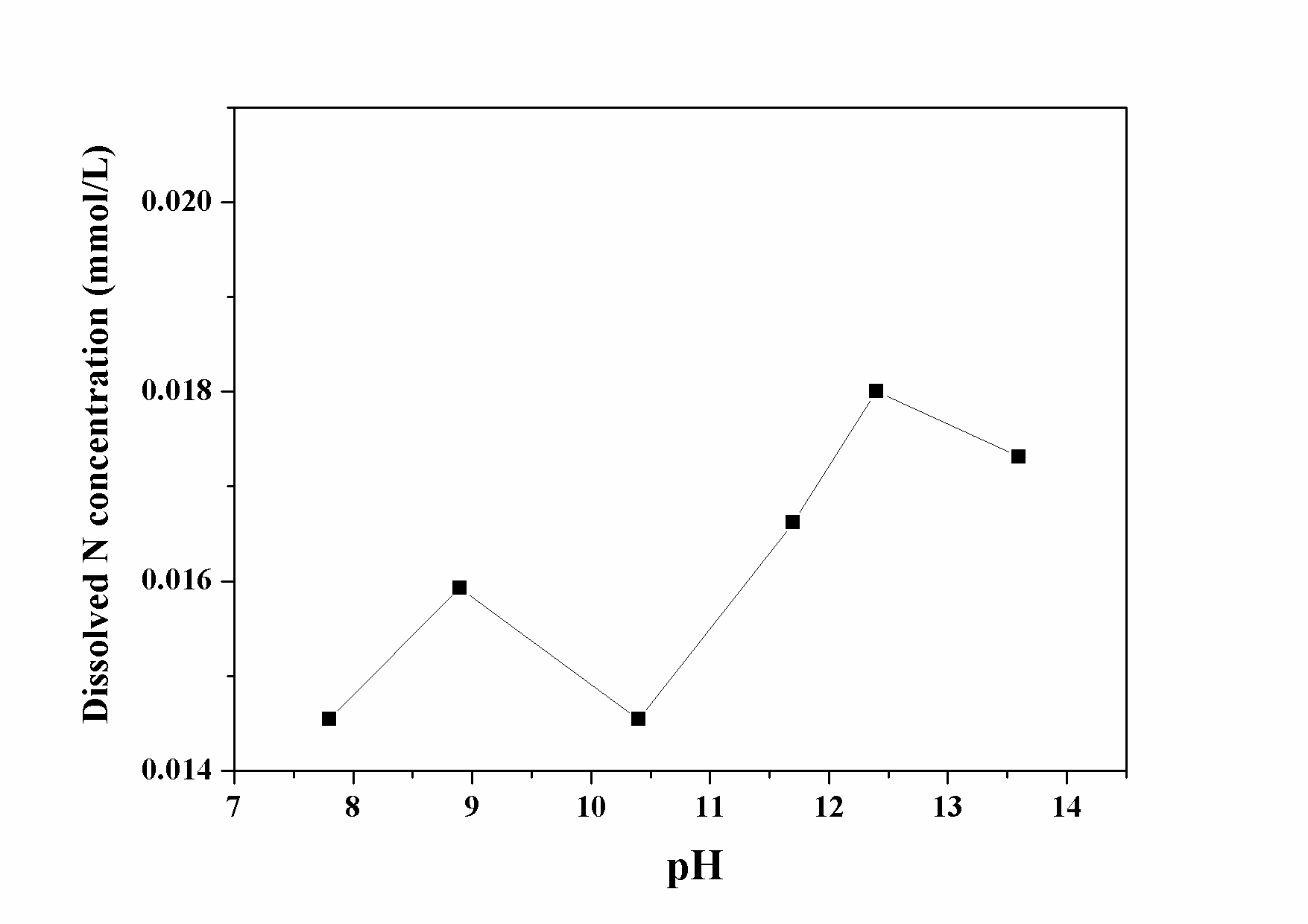


**(a)**

**(b)**

**S1 Fig (a) The absorbance as a function of molar concentrations of APTES, (b) The dissolved total nitrogen concentration under different pH values.**

**The chemical stability of APTES-Mt**

we have designed and conducted experiments to examine the hydrolyzation rate of APTES-Mt. We designed a simple and effective method to assess the stability of APTES-Mt. If the ether linkage (Si-O-Si, not ester linkage) is hydrolyzed, N element will dissolve into the solution. So the hydrolyzation rate is determined through measurement of the total nitrogen (TN) in the aqueous solution (in which APTES-Mt is suspended) since we have mature methods for TN determination in our laboratory.

We prepared APTES solution with concentrations of 5 mg/L-100 mg/L, and used K_2_S_2_O_8_ and H_2_SO_4_ to heat and digest the APTES solution, transferring the APTES-NH_2_ to NO_3_^-^. The TN determination method was used to determine the absorbance (A_220 nm_-2A_275 nm_) by UV-vis spectrophotometer. The absorbance as a function of molar concentrations of APTES (also molar concentrations of N) was shown in **S1 Figure (a)**.

Then the experiment testing the stability of APTES-Mt was performed. 0.05 g of APTES_4.0CEC_-Mt (with the maximum APTES loading) was added into a 50 mL flask containing 25 mL deionized water solution (2.0 g/L of APTES-Mt). The pH values (7-14) of the solution was adjusted by HCl (aq) or NaOH (aq). A blank flask (only 25 mL deionized water) was set as the control sample. The flasks were put into water-bathed at 30 ^o^C and agitated for 24 h, 200 rpm. After centrifugal separation, the TN concentration of supernate was determination. The results were shown in **S1 Fig (b)**. The maximum dissolved N concentration is 0.018 mmol/L, that is about 2.5 mg/L NH_2_-CH_2_-CH_2_-CH_2_-Si-(OH)_3_. In other word, 2.0 g APTES_4.0CEC_-Mt would release less than 2.5 mg silanes after 24 h of agitation. Therefore, the stability of APTES-Mt is satisfied.
