# Supplementary material for: Efficient Removal of Co2+ from Aqueous Solution by 3-Aminopropyltriethoxysilane Functionalized Montmorillonite with Enhanced Adsorption Capacity
Source: PLoS One. 2016 Jul 22;11(7):e0159802. doi: 10.1371/journal.pone.0159802 (PMC4957767; doi:10.1371/journal.pone.0159802)
Supplement: S1 Table — (DOC) [file pone.0159802.s002.doc]

**S1 Table.** **Infrared wavenumbers and assignments of Ca-Mt and APTES-Mts.**

| **Position/cm^-1^** | **Assignments** | **Position/cm^-1^** | **Assignments** |
| --- | --- | --- | --- |
| 3624 | Al-OH stretching of structure | 3432 | OH stretching of water |
| 1640 | OH deformation of water | 1088 | Si-O streching of structure |
| 1033 | Si-O streching | 914 | Al-Al-OH deformation of structure |
| 840 | Al-Mg-OH deformation of structure | 795 | Si-O flexing of structure |
| 624 | Si-O deformation of structure | 519 | Al-O-Si deformation of structure |
| 2925/2932/2933 | CH_2_ asymmetric stretching | 1507/1509/1512/1513 | N-H symmetric flexing |
| 1448/1450 | CH_3_ asymmetric flexing | 1414/1419 | C-H flexing |
| 694/695/697 | O-Si-O asymmetric flexing | 2316/2317 | N-H stretching |
| 2042/2088 | NH_3_^+^ asymmetric stretching | 1562 | NH_3_^+^ symmetric flexing |
