# Supplementary material for: Efficient Removal of Co2+ from Aqueous Solution by 3-Aminopropyltriethoxysilane Functionalized Montmorillonite with Enhanced Adsorption Capacity
Source: PLoS One. 2016 Jul 22;11(7):e0159802. doi: 10.1371/journal.pone.0159802 (PMC4957767; doi:10.1371/journal.pone.0159802)
Supplement: S2 Table — (DOC) [file pone.0159802.s003.doc]

**S2 Table. Comparison of adsorption capacity of Co^2+^** on various adsorbents.

| **Adsorbents** | **Adsorption capacity**  **(mg/g)** | **Initial concentration**  **(mg/L)** | **Equilibrium time** | **pH** | **Ionic strength** | **Temperature**  **(^o^C)** | **Reference** |
| --- | --- | --- | --- | --- | --- | --- | --- |
| APTES4.0-Mt | 41.37 | 100 | 8 h | 7 | 0.1 mol L^-1^ with KNO_3_ | 28 | this study |
| magnetite (Fe_3_O_4_) | 17.11 | 5.015 | 3 min | 6.9 | 0.01 mol L^-1^ NaNO_3_ | 22 | 1 |
| NiO | 200.6 | 20 | 16 h | 7.5 | 0 | 30 | 2 |
| clinoptilolite | 3.43 | 300 | 6 h | 5 | 0 | 20 | 3 |
| g-Al_2_O_3_ | 47.56 | 590 | 2 h | 5.5 | 0 | 28 | 4 |
| ETAS-10 | 59.83 | 1770 | 1 min | 4 | 0 | 28 | 5 |
| PET-TSC fibers | 78.08 | 150 | 50 min | 5 | 0 | 30 | 6 |
| palygorskite | 4.35 | 10 | 3 h | 6 | 0 | 35 | 7 |
| dried activated sludge | 9.164 | 59 | 2 h | 6 | 0 | 22 | 8 |
| oxidized carbon nanotube sheet | 16.19 | 100 | >70 h | 7 | 0 | 25 | 9 |
| modified magnetic chitosan chelating resin | 38 | 100 | 70 min | 5 | 0 | 28 | 10 |

**References:**

1. Uheida A, Salazar-Alvarez G, Björkman E, Zhang Y, Muhammed M (2006) Fe_3_O_4_ and gamma-Fe_2_O_3_ nanoparticles for the adsorption of Co^2+^ from aqueous solution. J Colloid Interface Sci. Journal of Colloid & Interface Science 298: 501-507.

2. Naeem A, Saddique MT, Mustafa S, Tasleem S, Shah KH, et al. (2009) Removal of Co^2+^ ions from aqueous solution by cation exchange sorption onto NiO. Journal of Hazardous Materials 172: 124-128.

3. Smičiklas I, Dimović S, Plećaš I (2007) Removal of Cs ^1+^ , Sr ^2+^ and Co ^2+^ from aqueous solutions by adsorption on natural clinoptilolite. Applied Clay Science 35: 139-144.

4. Bulbulian, Silvia (2013) Surface Characterization of gamma-Al2O3 Powders and Their Co^2+^;Adsorption Properties. International Journal of Applied Ceramic Technology 10: E295-E303.

5. Li W, Poirson A (2006) Adsorption behaviors of ETS-10 and its variant, ETAS-10 on the removal of heavy metals, Cu^2+^ , Co^2+^ , Mn^2+^ and Zn^2+^ from a waste water. Microporous & Mesoporous Materials 96: 157-167.

6. Monier M, Da. AL (2013) Modification and characterization of PET fibers for fast removal of Hg(II), Cu(II) and Co(II) metal ions from aqueous solutions. Journal of Hazardous Materials 250-251: 122-130.

7. He M, Zhu Y, Yang Y, Han B, Zhang Y (2011) Adsorption of cobalt(II) ions from aqueous solutions by palygorskite. Applied Clay Science 54: 292-296.

8. Frišták V, Pipíška M, Valovčiaková M, Lesný J, Rozložník M (2013) Monitoring 60 Co activity for the characterization of the sorption process of Co ^2+^ ions in municipal activated sludge. Journal of Radioanalytical & Nuclear Chemistry 299: 1607-1614.

9. Tofighy MA, Mohammadi T (2011) Adsorption of divalent heavy metal ions from water using carbon nanotube sheets. Journal of Hazardous Materials 185: 140-147.

10. Monier M, Ayad DM, Wei Y, Sarhan AA (2010) Adsorption of Cu(II), Co(II), and Ni(II) ions by modified magnetic chitosan chelating resin. Journal of Hazardous Materials 177: 962-970.
