# Supplementary material for: Efficient Removal of Co2+ from Aqueous Solution by 3-Aminopropyltriethoxysilane Functionalized Montmorillonite with Enhanced Adsorption Capacity
Source: PLoS One. 2016 Jul 22;11(7):e0159802. doi: 10.1371/journal.pone.0159802 (PMC4957767; doi:10.1371/journal.pone.0159802)
Supplement: S3 Table — (DOC) [file pone.0159802.s004.doc]

**S3 Table.** the raw data of adsorption of Co^2+^ in 30 ^o^C for better understanding of Fig 11.

| *C_0_* | *C_e_* | *q_e_* |
| --- | --- | --- |
| 8.64 | 0.01 | 4.32 |
| 29.9 | 0.01 | 14.95 |
| 47.2 | 0.04 | 23.58 |
| 69.72 | 0.097 | 34.81 |
| 87.15 | 0.586 | 43.28 |
| 90.02 | 0.99 | 44.5 |
